# Nickel platinum (Ni_*x*_Pt_1−*x*_) nanoalloy monodisperse particles without the core–shell structure by colloidal synthesis†

**DOI:** 10.1039/d0na00450b

**Published:** 2020-07-09

**Authors:** Cora Moreira Da Silva, Armelle Girard, Maxime Dufond, Frédéric Fossard, Amandine Andrieux-Ledier, Vincent Huc, Annick Loiseau

**Affiliations:** Laboratoire d'Étude des Microstructures, CNRS, ONERA, U. Paris-Saclay Châtillon 92322 France annick.loiseau@onera.fr; Université Versailles Saint-Quentin, U. Paris-Saclay Versailles 78035 France; Département Physique, Instrumentation, Environnement, Espace, ONERA, U. Paris-Saclay Châtillon 92322 France; Institut de Chimie Moléculaire et des Matériaux d'Orsay, CNRS, Paris Sud, U. Paris-Saclay Orsay 91045 France vincent.huc@universite-paris-saclay.fr

## Abstract

We report a new and versatile colloidal route towards the synthesis of nanoalloys with controlled size and chemical composition in the solid solution phase (without phase segregation such as core–shell structure or Janus structure) or chemical ordering. The principle of the procedure is based on the correlation between the oxidation–reduction potential of metal cations present in the precursors and the required synthesis temperature to nucleate particles without phase segregation. The procedure is demonstrated *via* the synthesis of Face Centered Cubic (FCC) Ni_*x*_Pt_1−*x*_ nanoparticles, which was elaborated by the co-reduction of nickel(ii) acetylacetonate and platinum(ii) acetylacetonate with 1,2-hexadecanediol in benzyl ether, using oleylamine and oleic acid as surfactants. The chemical composition and solid solution FCC structure of the nanoalloy are demonstrated by crosslinking imaging and chemical analysis using transmission electron microscopy and X-ray diffraction techniques. Whatever the chemical composition inspected, a systematic expansion of the lattice parameters is measured in relation to the respective bulk counterpart.

## Abstract 2

Versatile colloidal route towards the synthesis of nanoalloys with controlled size and chemical composition, based on the correlation between the oxidation–reduction potential of metal cations in the precursors and the synthesis temperature.

## Graphical abstract

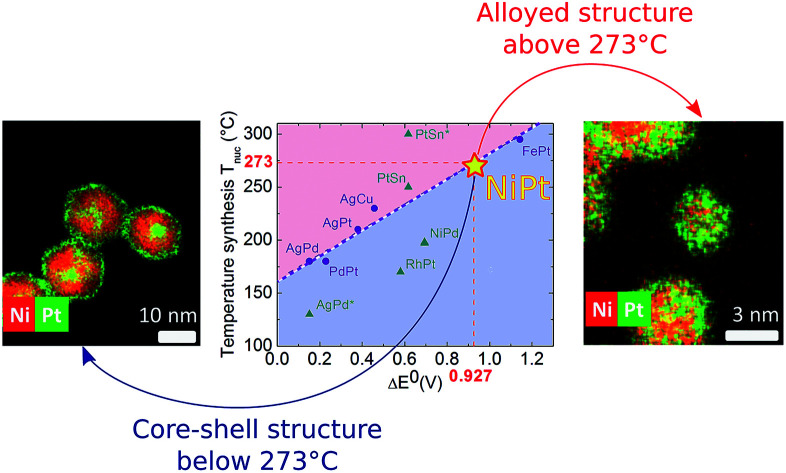

## 1 Introduction

Nanomaterials are widely studied in many scientific fields because of their specific reactivity due to their size.^1,2^ Metallic nanoparticles (NPs) have revolutionized the world of catalysis. Their reduced size, which maximizes their specific surface area, gives them a chemical reactivity different from their bulk counterpart.^1–3^ Furthermore, playing with nanoalloyed particles provides additional keys for tuning catalytic properties beyond size effects.^4–7^ This approach is today one of the most promising routes to the selective growth of Single Wall Carbon Nanotubes (SWCNTs).^8–12^ The main synthesis process of these nano-objects is chemical vapor deposition (CVD), based on the catalytic decomposition of an organic gas precursor at the surface of metallic nanoparticles. As their metallic or semi-conducting character directly depends on their diameter and chirality,^13^ controlling these structural parameters during the synthesis remains a hot challenge for the development of many applications. Most advanced modellings relate the chirality of SWCNTs to their growth mode and to the carbon content of the catalyst nanoparticles from which the nanotube grows.^10,14–16^

One way to control the carbon content is to play with the carbon solubility of the catalyst.^14–18^ Our approach is to tune it by nanoalloying the catalyst. To that aim, we have chosen to study Ni_*x*_Pt_1−*x*_ particles, with the composition ranging from pure Ni to pure Pt. Ni is known for its carbon solubility^19–21^ unlike platinum. The Ni–Pt bulk phase diagram indicates the stability of the solid solution regardless of the alloy concentration in all the temperature ranges visited to synthesize SWCNTs. Platinum is expected to modulate the catalytic activity of Ni by varying the carbon solubility in the NPs. To study the impact of this parameter on CNT growth (ongoing), synthesis of Ni, Ni_3_Pt, NiPt, NiPt_3_ and Pt (compositions defined in the bulk)^22,23^ homogeneous nanoalloys with identical controlled size and shape is required. Towards this goal, NP synthesis can be achieved *via* physical or chemical routes.

Physical routes, such as magnetron ion sputtering,^24^ molecular beam epitaxy (MBE)^25^ and pulsed laser deposition (PLD)^26^ are essentially based on the production of metal vapors and their condensation on a substrate surface. The resulting particles are of high purity and small size, monodispersed and their composition is easily controllable through the metal composition of the vapor. Despite these numerous advantages, these NPs are difficult to handle due to their size and are easily oxidized in air. Surface treatment (protective layer) is thus frequently necessary. To control their size, alloying or core–shell state and crystallographic structure, annealing is often necessary but can lead to undesired chemical modifications, coalescence and Ostwald ripening, resulting in polydispersed NPs.

Chemical routes encompass a large variety of techniques such as solvothermal,^27,28^ colloidal,^29,30^ sonochemical^31,32^ synthesis or use of well-defined pre-catalysts such as prussian blue analogs.^33,34^ Among them, the colloidal route is highly attractive for our purpose as it is an efficient and reliable route to obtain NPs with tunable size, shape and chemical composition.^35^ The principle of colloidal synthesis consists in the co-reduction of metal precursors assisted by a reducing agent, in the presence of surfactants in an organic medium, at a given temperature. Reduced metal atoms react with each other to form alloy particles, coated by surfactant molecules. The role of the long carbon chains attached to the NP surface is to block the growth of the NPs,^36^ set their size and stabilize them by minimizing surface energies.^2,37,38^ Size tunability is achieved by adjusting the metallic precursor : surfactant ratio.^39^ The organic coating makes the NPs soluble in organic solvents and protects them from oxidation. The nature of the generated particles depends on several parameters such as (i) the inherent reactivity of the reagents used, (ii) the quantity of reagents used, (iii) temperature and (iv) reaction time.

In this study we demonstrate the efficiency of the colloidal route for the synthesis of alloyed nickel–platinum NPs, without phase segregation or a core–shell structure, with controlled chemical composition from pure Ni to pure Pt and controlled size below 5 nm.

## 2 Chemical synthesis route to nanoalloyed particles

**Fig. 1:**
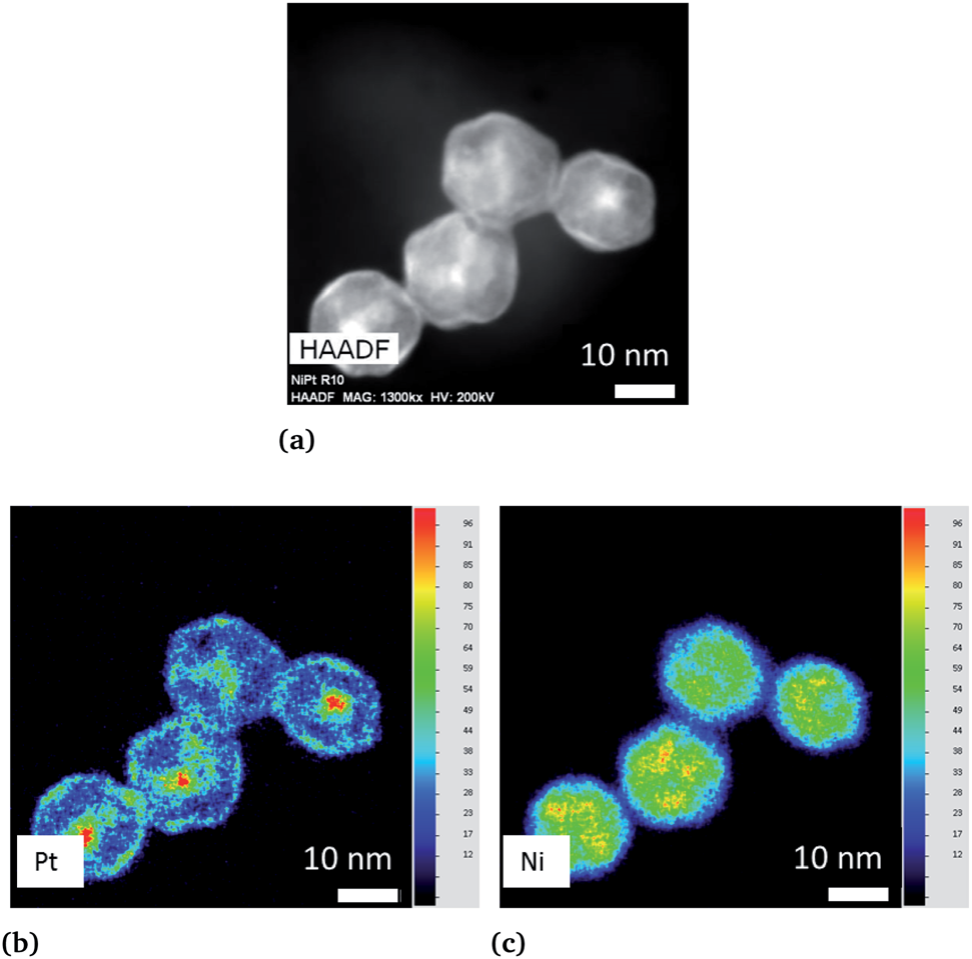
Ni_3_Pt colloidal synthesis performed at 225 °C. (a) STEM-HAADF image and chemical mappings using EDX mode with the concentration scale demonstrate that (b) Pt (L_α_ line) is predominantly located in the core and (c) Ni (K_α_ line) mainly in the shell.

We first applied the procedure used in previous studies for the colloidal synthesis of Ni_*x*_Pt_1−*x*_ nanoalloys, which involves simultaneous reduction reactions of Pt(acac)_2_ and Ni(acac)_2_ at 220–230 °C. In agreement with the literature,^30,39–43^ we obtained under these conditions NPs with either a core–shell Pt@Ni (Pt core surrounded by Ni shell) structure or a platinum concentration gradient from the NP center to its surface as shown in the images of Fig. 1 (and Fig. S1 of the ESI†). In Fig. 1a, recorded with high-angle annular dark-field imaging (HAADF) in scanning transmission electron microscopy (STEM) mode, the contrast in the image is related to *Z*, the atomic number difference between Pt (78) and Ni (28), with brighter zones in the core of the NPs indicating a Pt-rich concentration. The core–shell structure is confirmed from chemical mappings using energy-dispersive X-ray spectroscopy (EDX) (see Fig. 1b, c and EDX spectra analysis of the four NPs of Fig. 1, and Fig. S2 of the ESI†). The signal from Ni atoms is constantly depressed in areas where Pt atoms are present, reflecting a clear chemical segregation. In Fig. 1b, increase of the Pt signal is noted on the NP surface. The observed images are projections, but if one facet of the NP is parallel to the electron beam, there is strengthening due to the thickness and not the chemical composition (see intensity profiles Fig. S3 of the ESI†).

**Fig. 2:**
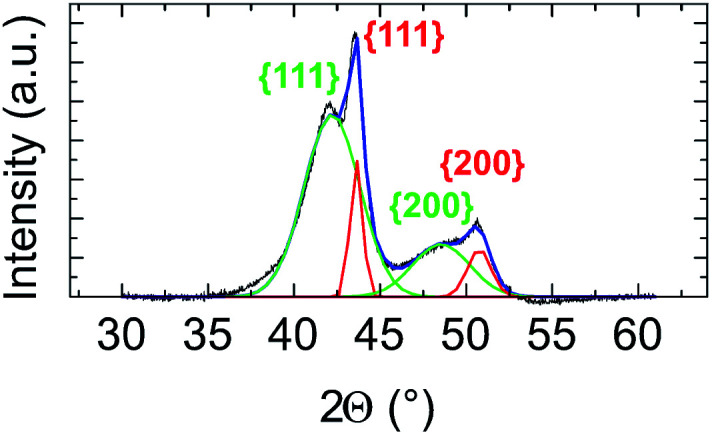
Ni_3_Pt (75 : 25) elaborated at 225 °C core–shell PXRD patterns, deconvolution of the different peaks (in blue) and indexing of peaks assuming that the core is the NiPt solid solution in green and the shell, the Ni_3_Pt solid solution in red.

In Fig. 2, the crystal structure is verified by powder X-ray diffraction (PXRD). The deconvolution of the PXRD pattern shows the presence of two FCC phases. Indexing of XRD peaks demonstrates the coexistence of an FCC disordered NiPt phase (in green) and FCC disordered Ni_3_Pt (in red), which is proof of a Pt concentration gradient within NPs from their core to their surface. The respective lattice parameters are listed in Table S1 (ESI†). Furthermore, thanks to Scherrer formula applied to (111) peaks, we determined two specific sizes, the first one equal to 2.7 nm (NiPt phase), which can be assigned to the core of the NPs and the second one equal to 9.9 nm (Ni_3_Pt phase), which corresponds to the shell morphology. This result is in agreement with EDX analysis (see Fig. 1b and c).

**Fig. 3:**
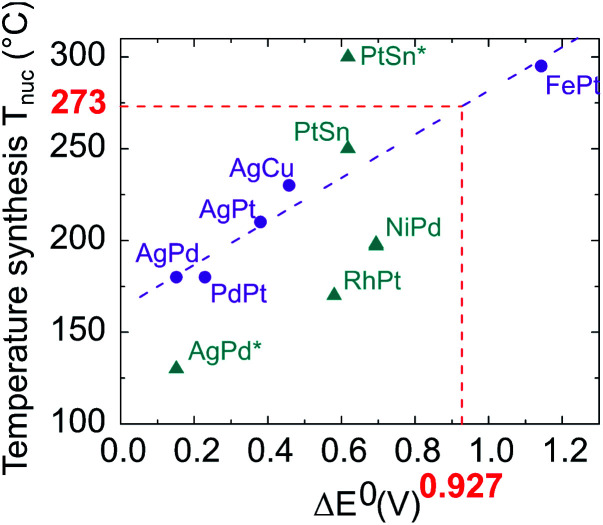
Proposed temperature synthesis for alloying different elements *versus* the difference in their reduction potentials Δ*E*^0^. Purple circles: acetyl or acetylacetonate metallic precursor family (AgPd,^49^ PdPt,^50^ AgPt (unpublished work), AgCu,^51^ FePt^52^). Blue triangles: metallic precursors with different natures; nitrate (AgPd*^53^), chlorate (RhPt,^54^ PtSn solid solution and PtSn* ordered compound^55^), sulfate (NiPd^56^).The value of Δ*E*^0^ equal to 0.927 depicted in red corresponds to the NiPt case.

As these results were far from our objective, we paid particular attention to identifying the effects responsible for the chemical segregation observed. Our analysis is that the segregation is first caused by the difference between the standard reduction potentials of platinum in the oxidation state +II in Pt(acac)_2_ (*E*^0^(Pt^II^/Pt^0^) = +1.18 V) and of nickel +II as well in Ni(acac)_2_ (*E*^0^(Ni^II^/Ni^0^) = −0.253 V), which generates different reduction kinetics.^44–46^ It is known that the higher the difference between the oxidation–reduction potential and the potential of the standard hydrogen electrode (thermodynamic scale of redox potentials), the faster the kinetics of the reduction reaction will be. Ni(acac)_2_ thermally decomposes at ≈230 °C (ref. 39) while Pt(acac)_2_ at ≈160 °C.^47^ As a consequence, in the same medium at temperatures around 220–230 °C, the Pt precursor is decomposed and reduced faster and before Ni, resulting in the formation of a Pt *nucleus*. Then, once Ni reduction occurs, Ni atoms settle on the Pt *nuclei* surface, forming a shell. This phenomenon has already been observed by Chen *et al.* with the NiRu system,^48^ who overcame it by using a strong reducing agent. In addition, we identified temperature as the second key parameter. Temperature is a well-known parameter in bulk alloy synthesis. Solid solutions in alloys such as Ni–Pt can only be obtained if annealing treatments at a sufficiently high temperature are carried out, for atomic diffusion to be effective. On this basis, we have compiled in Fig. 3 literature data on the synthesis of various bimetallic nanoalloys through the colloidal route with a demonstrated solid solution phase (2–10 nm in diameter) and plotted their synthesis temperature against the standard reduction potential difference (in absolute values) between the two components of the nanoalloy. As a general feature, the higher the difference in the redox potentials Δ*E*^0^, the higher the temperature necessary for nucleating a solid solution, *T*_nuc_. The nature of the ligands present in the metallic precursors is also found to impact *T*_nuc_. The groups bonded to metal cations have indeed different inductive effects depending on their nature (acetylacetonate, sulfate, nitrate, *etc.*) and induce different apparent charges on the cation, involving a variation in its redox potential.

The synthesis depends on three variables: the apparent redox potential difference of the metal cations in the precursors, the reducing power of the reductant and the temperature. Two parameters are fixed: the apparent redox potential induced by groups bonded to metal cations and the reducing force of the reductant; considering only data from synthesis with acetyl or acetylacetonate metallic precursors reduced by a mild reducing agent, which we use in our procedure (purple dots in Fig. 3), a linear dependence emerges between Δ*E*^0^ and *T*_nuc_, as outlined by the purple fitting dashed line.

**Fig. 4:**
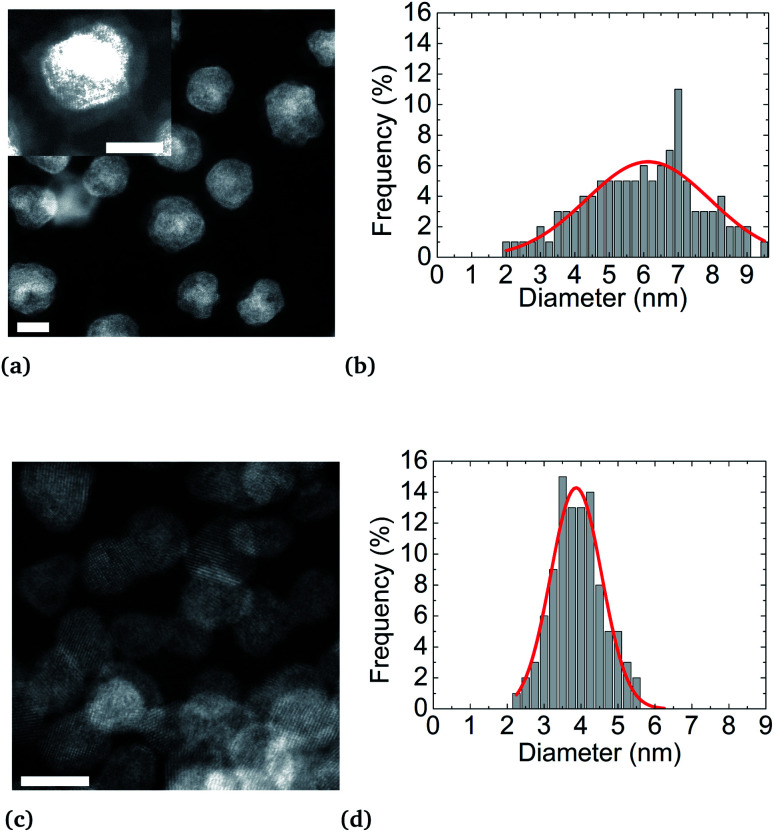
STEM-HAADF micrographies and size distribution histograms of Ni_3_Pt NPs elaborated at (a and b) 200 °C showing a core–shell structure and an observable organic layer surrounding the particle in the magnified image in the inset and at (c and d) 280 °C showing alloyed particles (scale bar = 5 nm).

Using this linear fit, we infer that the synthesis temperature of a Ni–Pt nanoalloy should be close to 270 °C, knowing that the difference between the standard reduction potentials of platinum and nickel is equal to Δ*E*^0^ = 0.927 V. Such synthesis conditions are achievable since the decomposition temperature of Ni(acac)_2_ and Pt(acac)_2_ is below this temperature. We thus decided to increase the reaction temperature. However, to prevent the formation of Pt *nuclei*, it is necessary to introduce both metallic precursors at the same time, at the working temperature. A “hot injection” process has thus been designed, where solutions of both metallic precursors are rapidly injected in the reaction media. On this basis we proceeded as follows. We performed two new syntheses of Ni_3_Pt nanoparticles, one at 200 °C in 10 mL of benzyl ether (see Fig. 4a) and the other at 280 °C in a large quantity of solvent (70 mL), to limit the temperature drop when injecting “cold” precursors (see Fig. 4b).

STEM-HAADF analyses reveal a core (Pt)–shell (Ni) structure for the nanoparticles synthesized at 200 °C. However, as expected, synthesis at 280 °C results in nanoparticles with a monotonous HAADF contrast, indicating an alloyed state (Fig S4b of ESI†). Furthermore, as already mentioned,^57^ higher synthesis temperature has the effect of activating particle nucleation, resulting in an improvement of the size monodispersity and narrowness peaking at *d* = (3.9 ± 0.7) nm in diameter against *d* = (6.2 ± 3.5) nm for the synthesis at 200 °C, for the same reaction yields. After NP synthesis and their centrifugation, the supernatant obtained is colorless while the metal precursors are colored (green for Ni(acac)_2_ and yellow for Pt(acac)_2_). This evidences the full consumption of metal precursors during the synthesis. Moreover, regardless of their state (alloy or a core–shell), NPs are coated by an organic layer. This coating is shown in the inset of Fig. 4a. The dense coating is approximately 1 nm thick and uniformly covers the NPs. Oleylamine CH_3_(CH_2_)_7_CH = CH(CH_2_)_8_NH_2_ and oleic acid CH_3_(CH_2_)_7_CH = CH(CH_2_)_7_COOH molecules are both 2 nm (ref. 58 and 59) long, in their so-called “linear” configurations. These molecules can fold back at their double bond, located close to their center, thus reducing their apparent length to about 1 nm. This covering may be due to NPs drying on the TEM copper grid. We can deduce that an effective uniform surfactant monolayer hangs on the NP surface.

## 3 Results and discussion

**Fig. 5:**
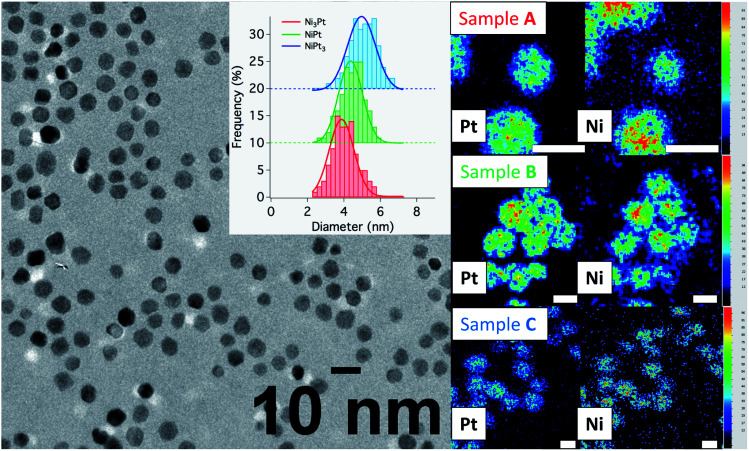
(Left) Bright-field micrography of sample C representative of the NP dispersion for all samples studied and histograms (in the inset) showing particle size distributions in samples A (in red), B (in green) and C (in blue). (Right) EDX chemical mappings showing the spatial correlation between Ni and Pt on individual NPs in sample A (top), B (middle), C (bottom) (scale bar = 5 nm and concentration scale from 0 (black) to 100% (red)).

Next, a series of samples with targeted compositions, Ni_3_Pt, NiPt and NiPt_3_, were synthesized by adjusting Ni : Pt ratios and processing the hot injection at 280 °C, followed by a temperature drop at 275 °C. In the following discussion, the different samples are named A for Ni-rich, B for equiatomic composition and C for Pt-rich NPs. Their characterization by TEM techniques and PXRD is presented in Table 1, Fig. 5 and 7, with details in the ESI (Fig. S5, S6 and Table S1†).

**Table 1:** Average chemical atomic composition and particle size deduced from EDX and TEM image analyses respectively

Ni_*x*_Pt_1−*x*_	*x*	Particle size (nm)
A	0.74 ± 0.04	3.9 ± 0.7
B	0.56 ± 0.06	4.1 ± 0.7
C	0.32 ± 0.07	4.8 ± 0.7

**Fig. 6:**
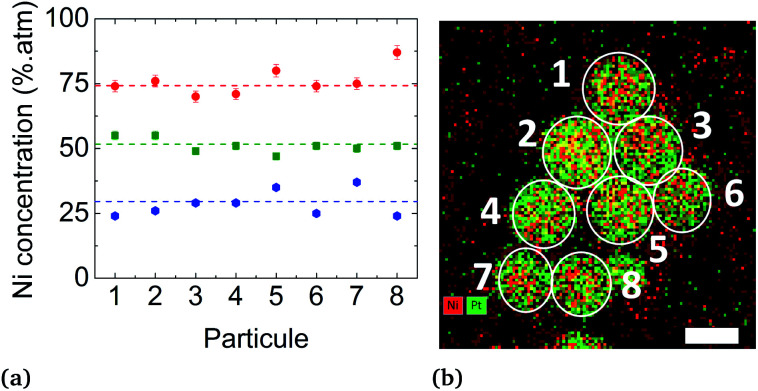
(a) Chemical composition distribution extracted from EDX chemical mappings recorded on isolated NPs in samples A (in red), B (in green) and C (in blue). (b) Example of a chemical mapping sampling of 8 NPs in sample B with Ni in red and Pt in green (scale bar = 5 nm).

Conventional TEM images of as-synthesized NPs show crystallites with spherical shape and an average diameter around 4 nm for all of them. EDX measurements using the K_α_ line of Ni and L_α_ line of Pt have been performed on different areas of the TEM grids in a statistical way (Fig. 5). Average values of the chemical compositions extracted from the spectra indicate that chemical compositions are very close to the target stoichiometries 1 : 3, 1 : 1 and 3 : 1 (see Table 1), due to the total precursor consumption during the reaction. Furthermore, whatever the targeted composition, EDX chemical mappings display very little composition variation from a NP to another as shown in Fig. 6, demonstrating the capability of our synthesis procedure in controlling both the average and the dispersion of the NP composition.

The solid solution state of the particles is attested by both EDX and diffraction analyses. EDX chemical mappings presented in Fig. 5, 6 and EDX spectra in Fig. S5 (ESI)† show that both Ni and Pt species are uniformly spatially distributed in the volume and at the surface of the particles in all cases. In addition, this technique shows the absence of an oxide layer at the NP surface due to the homogeneous protective organic layer provided by the surfactants.

**Fig. 7:**
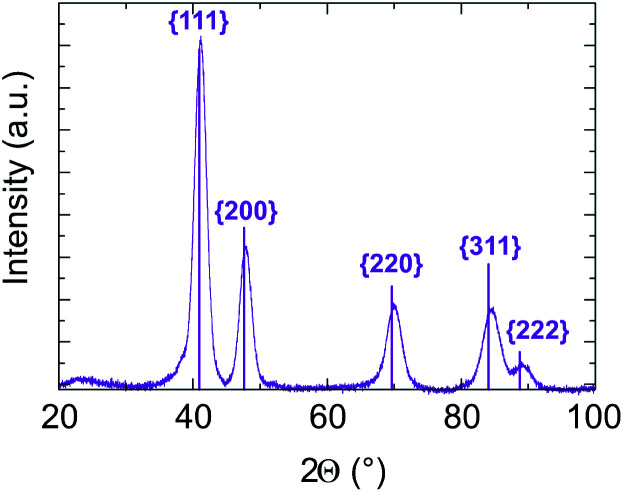
Sample B PXRD pattern analysed assuming an FCC structure with the lattice parameter equal to (0.380 ± 0.003) nm. The crystalline domain size is measured from the peak width to be equal to (4.5 ± 0.5) nm. The diffraction peak indexing related to the theoretical NiPt FCC structure.

In addition, in all cases, electron diffraction and PXRD (see Fig. S6 and Table 1 of ESI†) reveal that the particles are crystallized in a disordered FCC structure. No chemical order on the FCC lattice was observed, which is expected to be the stable state at equilibrium in the bulk system. As an example, PXRD data for sample B with targeted equiatomic composition are presented in Fig. 7. Diffraction peaks are well identified as being related to (111), (200), (220), (311) and (222) of the NiPt FCC disordered structure. The lattice parameter is calculated to be (0.380 ± 0.003) nm and the crystalline domain size is measured to be (4.5 ± 0.5) nm which is in good agreement with its apparent size in TEM images (Table 1). Therefore, the as-obtained NPs seem to be ≪one grain≫ alloy nanoparticles.

**Fig. 8:**
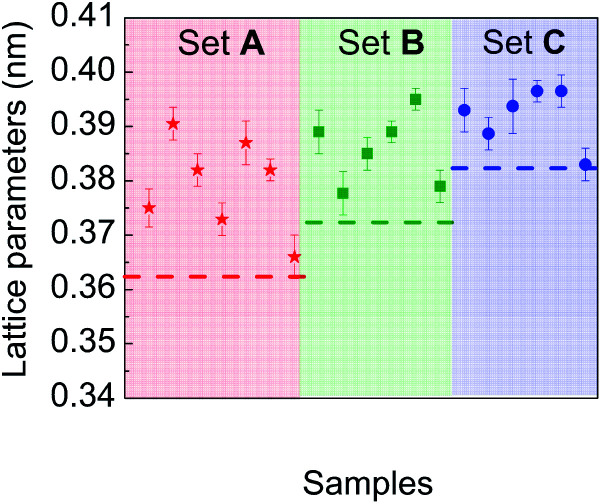
Lattice parameters measured from electron diffraction patterns on different sets of particles in samples A, B and C compared to reference values in the corresponding bulk materials (dashed straight line).

Particular attention was paid to comparing the measured lattice parameters with those of the corresponding bulk materials, for each chemical composition experimentally investigated. Fig. 8 is a plot of lattice parameter data deduced from electron diffraction patterns recorded on different sets of particles in samples A, B and C. Their comparison with reference data of the corresponding bulk materials with the same average composition shows a systematic expansion of the lattice parameters in the NPs whatever their composition.

**Fig. 9:**
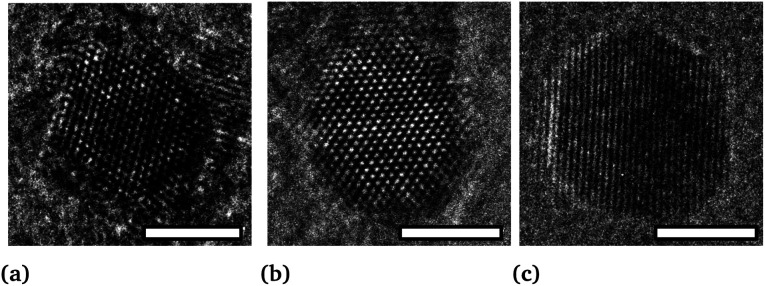
Nanoparticle HRTEM micrographies of sample (a) A, (b) B and (c) C (scale bar = 3 nm).

This expansion is verified by HRTEM analysis. The *d*-spacing between the planes (111) of particles, shown in Fig. 9, are equal to *d*_111_ (A) = 0.219 nm, *d*_111_ (B) = 0.225 nm and *d*_111_ (C) = 0.226 nm, corresponding to lattice parameters 0.379 nm, 0.389 nm and 0.391 nm, respectively. Moreover, it is important to note that there are no defects in the NPs (such as dislocations) or crystal twinning that could explain this lattice parameter expansion.^60^

This expansion is also confirmed by XRD on sample B (Fig. 7). Usually, contraction of lattice parameters is observed at the nanoscale due to surface strains.^61–63^ Here, surface stress is compensated by the presence of surfactants (to our knowledge, there is no study on the dependence of the lattice parameters on the surfactants covering nanoparticles by the colloidal synthesis route).

**Fig. 10:**
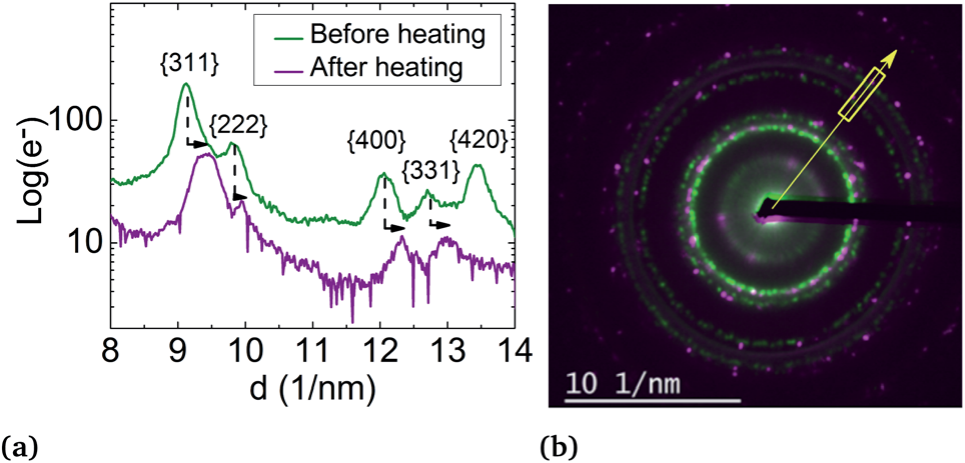
Effect of heating on the lattice parameter of a set of particles in sample B: (a) intensity profiles of electron diffraction peaks before heating (green line) and after heating (purple line) showing the peak shift. (b) Superimposition of electron diffraction before heating (in green) and after heating (in purple), showing contraction of the lattice from *a* = (0.373 ± 0.003) nm to *a* = (0.364 ± 0.003) nm. The direction of the intensity profile plot is shown by the yellow arrow. The yellow box corresponds to the range over which the intensity profile is plotted.

But, in order to capture the influence of surfactants on the lattice parameters, sample A NPs (Ni_3_Pt targeted composition) with *a* = (0.373 ± 0.003) nm were deposited on a SiN TEM grid covered by multi-walled carbon nanotubes (MWCNTs). MWCNTs are known for their high thermal conductivity and stability at high temperature,^64^ properties useful for our experiment. The NPs were heated *in situ* in ZEISS-LIBRA 200 MC microscope, in vacuum. Thanks to the heating holder, NPs were heated at 800 °C (≈39 °C min^−1^) and cooled down to room temperature (≈46 °C min^−1^). We have observed experimentally that when the temperature rose rapidly, the surfactants desorbed, pyrolyzed and not polymerized (see Fig. S7 in the ESI†). In addition, the temperature was decreased to avoid the incorporation of carbon from the surfactants into the NPs during the descent. Lattice parameter evolution is monitored by electron diffraction. In Fig. 8, electron diffraction patterns before and after the temperature increase are compared at room temperature, a decrease of the lattice parameter around 2.5% is observed (Fig. 10).

The lattice parameter decreases up to the bulk's value and not below, as provided in the literature. However, this can also be explained by the residual presence of surfactants on the NP surface or by the diffusion of carbon atoms in the particles readily solubilized by the nickel.

This result shows the first evidence of the influence of surfactants on lattice parameters. A detailed study of the impact of the surfactant nature *versus* metallic atoms on the expansion of the lattice parameter is underway.

## 4 Experimental procedure

### 4.1 Ni_*x*_Pt_1−*x*_ synthesis

To prevent contamination, all laboratory glassware was washed with *aqua regia* (nitric acid : hydrochloric acid 1 : 3) for (at least) five hours and rinsed with large amounts of distilled water, dichloromethane (DCM) and acetone. Syntheses were carried out under an argon blanket. Commercial reagents were used without purification. Typically, to obtain spherical Ni_3_Pt nanoparticles with 4 nm in size, 70 mL of benzyl ether (BE) (Sigma Aldrich, 99%), 1.22 mmol of oleylamine (OAm) (Acros Organics, 80–90%) and 1.26 mmol of oleic acid (OAc) (Acros Organics, 80–90%) were added to a 100 mL round-bottom flask with a PTFE coated magnetic stir bar. The mixture was purged by 3 vacuum/argon cycles and heated for 10 min at 100 °C, to remove any trace of water and prevent particle oxidation. After 10 min, the temperature was raised and kept at 280 °C. At this temperature, a solution of 0.33 mmol of Ni(acac)_2_ (Aldrich, 95%), 0.075 mmol of HDiol (TCI, 98%), 0.61 mmol of OAm, 0.63 mmol of OAc dissolved in a minimal amount of BE (1.3 mL) and a solution of 0.17 mmol of Pt(acac)_2_, 0.075 mmol of HDiol, 0.61 mmol of OAm, and 0.63 mmol of OAc dissolved in 1.3 mL of BE were simultaneously and quickly added (using two different syringes at the same time) to the round-bottom flask, under vigorous stirring. The solution turned instantly black, which was proof of NP nucleation. The suspension was kept at 280 °C for 2 min before being cooled down to room temperature under an argon blanket. The suspension was purified by adding 20 mL of ethanol (EtOH) for 10 mL of the mixture and was centrifuged at 10 000 rpm for 20 min. The supernatant was separated and the precipitate was washed again with EtOH and centrifuged. The precipitate was redispersed in 30 mL of DCM (few drops of OAc might be added to favor its redispersion). By controlling the Ni : Pt initial ratio during the synthesis, this method provides Ni_3_Pt, NiPt and NiPt_3_ NPs. All syntheses were performed at a constant total metal concentration equal to 0.5 mmol, divided between the Ni and Pt precursors to obtain Ni_3_Pt (3 : 1), NiPt (1 : 1) and NiPt_3_ (1 : 3).

### 4.2 Nanoparticle characterization

As-synthesized NPs were characterized by the Transmission Electron Microscopy (TEM) technique. A drop of colloidal suspension was deposited and dried on a copper TEM grid. Size distribution was measured using FEI-CM 20 TEM (200 kV) and JEOL USA JEM-1400 (120 kV) TEM instruments. A population of 500 particles in 5 distinct zones on the TEM grid was analysed and counted with Image J software. High-Resolution Transmission Electron Microscopy (HRTEM), High-Angle Annular Dark-Field imaging (STEM-HAADF) and electron diffraction (ED) were performed on a ZEISS-LIBRA 200 MC TEM to confirm the alloy state of the NPs. Chemical composition was also confirmed using Energy-Dispersive X-ray spectroscopy (EDX) (diode Brüker) using the NiPt bulk reference alloy. To support the results, UHRSTEM and chemical mapping were performed using a Titan G2 Cs-corrected FEI TEM operating at 200 kV on individual particles. The crystal structure of the core–shell and the alloyed sample were confirmed by X-ray diffraction (XRD) using Cu-Kα rays and 2*θ* scattering angle between 20° and 100°, in order to validate the TEM nanoparticle characterization protocol.

## 5 Conclusion

In this work, we have developed a new colloidal procedure for the synthesis of Ni_*x*_Pt_1−*x*_ nanoalloys with controlled size (nearly 4 nm) and chemical composition. Our study demonstrates the versatility of this synthesis method along with the key role of temperature synthesis in true alloy formation as a function of the redox potentials of the constituent elements. The chemical composition and solid solution FCC structure of the nanoalloys are demonstrated, over the whole range of chemical compositions investigated, by combining different TEM imaging modes, electron and X-ray diffraction techniques as well as EDX spectroscopy and mappings. We already checked the suitability of these new catalyst particles for the successive CVD synthesis of SWCNTs thanks to preliminary tests. A detailed study of the impact of the alloy composition on the structural characteristics of these SWCNTs is underway. Furthermore, this new synthesis procedure opens new avenues for catalysis, which are presently under investigation. It can be applied to a wide range of metallic alloys. Finally, we obtained the first evidence that surfactants capping the particles may impact their lattice parameters. Systematic measurements combined with *in situ* experiments are underway to clarify the influence of the surfactant on such disordered NPs.

## Conflicts of interest

There are no conflicts to declare.

## Supplementary Material

NA-002-D0NA00450B-s001
