# Supplementary material for: Nickel platinum (NixPt1−x) nanoalloy monodisperse particles without the core–shell structure by colloidal synthesis
Source: Nanoscale Adv. 2020 Jul 9;2(9):3882–9. doi: 10.1039/d0na00450b (PMC9417886; doi:10.1039/d0na00450b)
Supplement: NA-002-D0NA00450B-s001 [file NA-002-D0NA00450B-s001.pdf]

Electronic Supplementary Material (ESI) for Nanoscale Advances.
This journal is © The Royal Society of Chemistry 2020

Electronic Supplementary Material

Ni_xPt_{1-x} Nanoalloy Monodisperse Particles Without Core-shell Structure by Colloidal Synthesis

Cora Moreira Da Silva,[*a], Armelle Girard,[a,b] Maxime Dufond,[a,‡] Frédéric Fossard,[a] Amandine Andrieux-Ledier,[c] Vincent Huc[d] and Annick Loiseau[a]

[a] Laboratoire d'Etude des Microstructures, CNRS, ONERA, U. Paris-Saclay, Châtillon, 92322, France
[b] Université Versailles Saint-Quentin, U. Paris-Saclay, Versailles, 78035, France
[c] Institut de Chimie Moléculaire et des Matériaux d'Orsay, CNRS, Paris Sud, U. Paris-Saclay ; Orsay, 91045, France
[d] Département Physique, Instrumentation, Environnement, Espace, ONERA, U. Paris-Saclay, Châtillon, 92322, France
[‡] Present address : Centre Interdisciplinaire de Nanoscience de Marseille, CNRS, U. Aix-Marseille, CNRS, Aix-Marseille, 13009, France

1. Analyses of core-shell Ni_3Pt nanoparticles

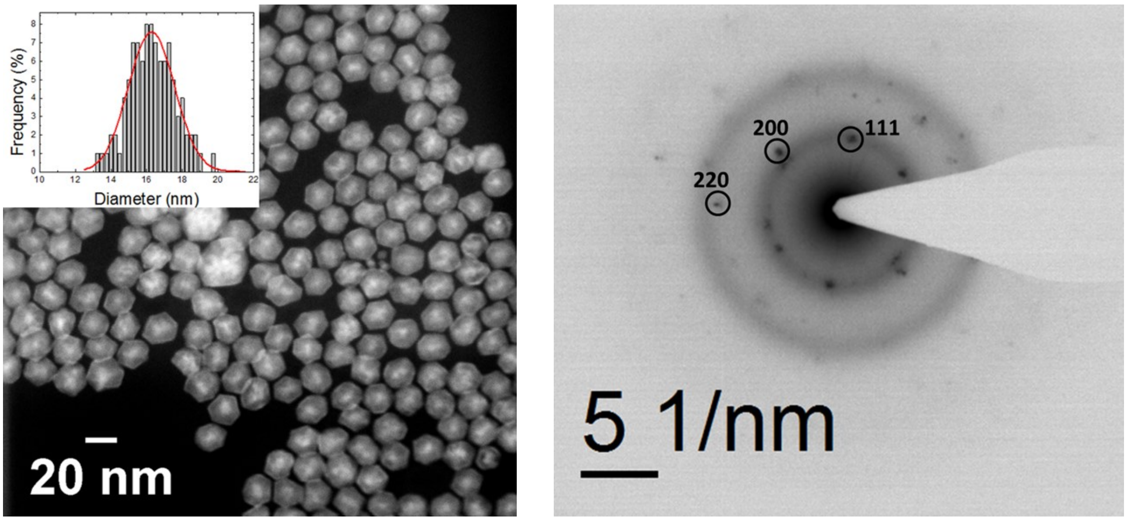

Fig. S1: **Left** HAADF-STEM image of core-shell Pt@Ni NPs with d = (16 ± 3) nm and the size distribution histogram. **Right** NPs electronic diffraction clearly shows 111, 200 and 220 peaks related to a FCC lattice parameter a = (0.369 ± 0.007) nm. Other peaks are observable but they are diffuse due to the core-shell structure.

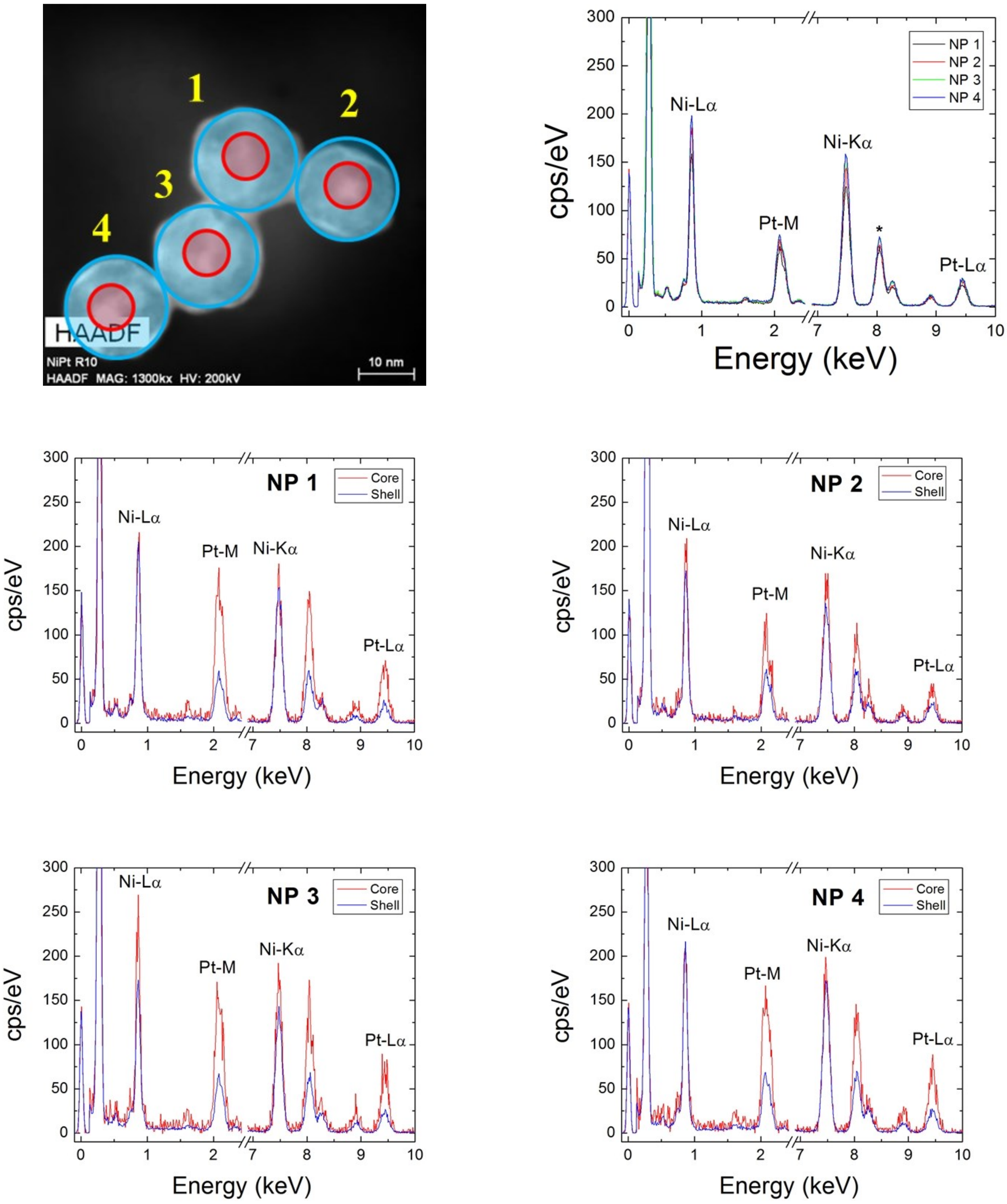

Fig. S2: Analysis of EDX spectra of the 4 NPs presented in Fig. 1.

Top HAADF image of the four analyzed NP labelled 1-4. Their core is highlighted in red and this shell in blue and comparison of the 4 spectra recorded on each NP.

The EDX spectra comparison of the 4 isolated NPs indicates an identical chemical composition near 87 % in nickel against 13 % in platinum (atomic %).

Bottom comparison for each particle of EDX spectra recorded from the core (red curve) and from this shell (blue curve).The chemical composition comparison between the core and the shell in isolated particles (see in Fig. 1) illustrates the Pt-rich core against Ni-rich shell.

* The copper peaks at 8.040 keV is due to the TEM grid.

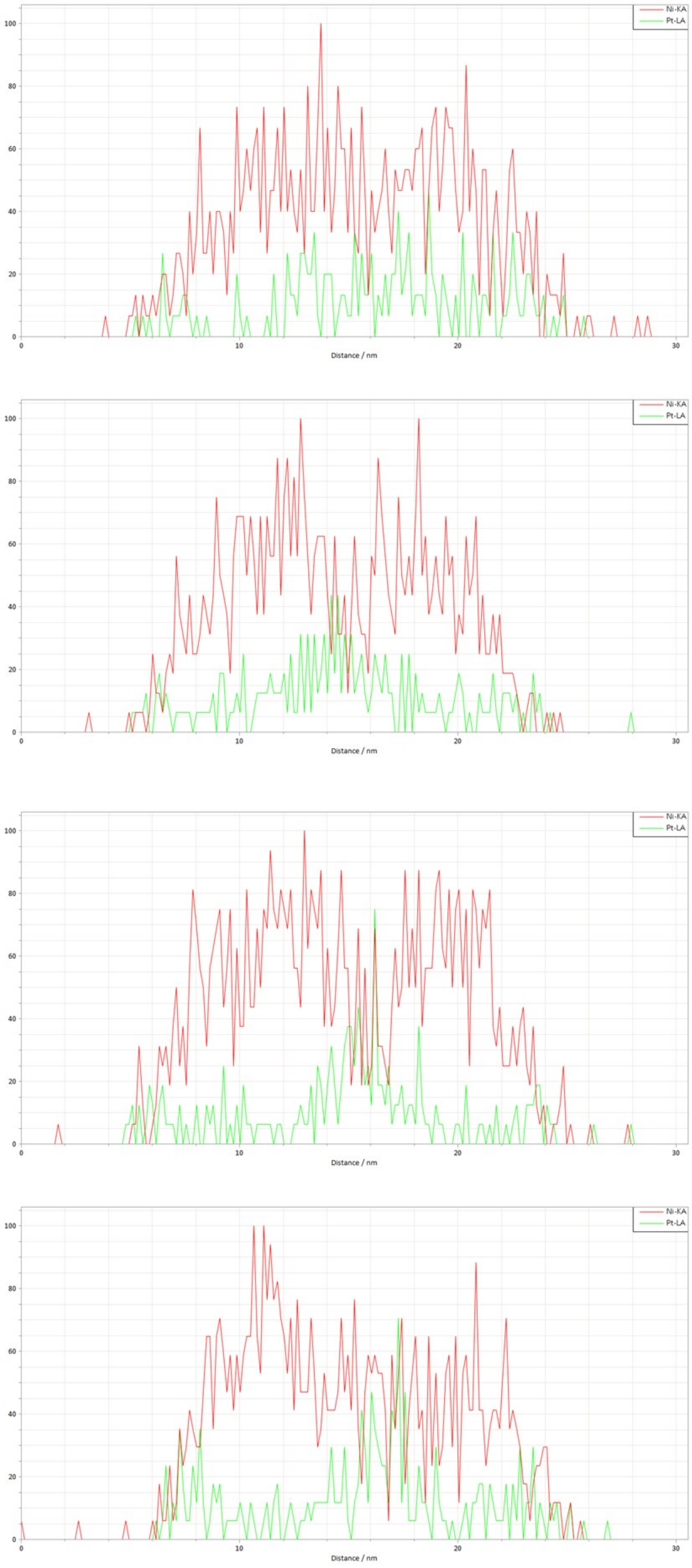

Fig. S3: Ni (in red) and Pt (in green) intensity profiles along the NPs shown in Fig. 1 and S2. At the edge of the particle, the amount of Pt varies a little, additionally it is in the same

quantity as for Ni. On the contrary, in the NPs center, a strong signal from Pt and a weak signal from Ni are observed.

2. Analyses of core-shell Ni_3Pt nanoparticles

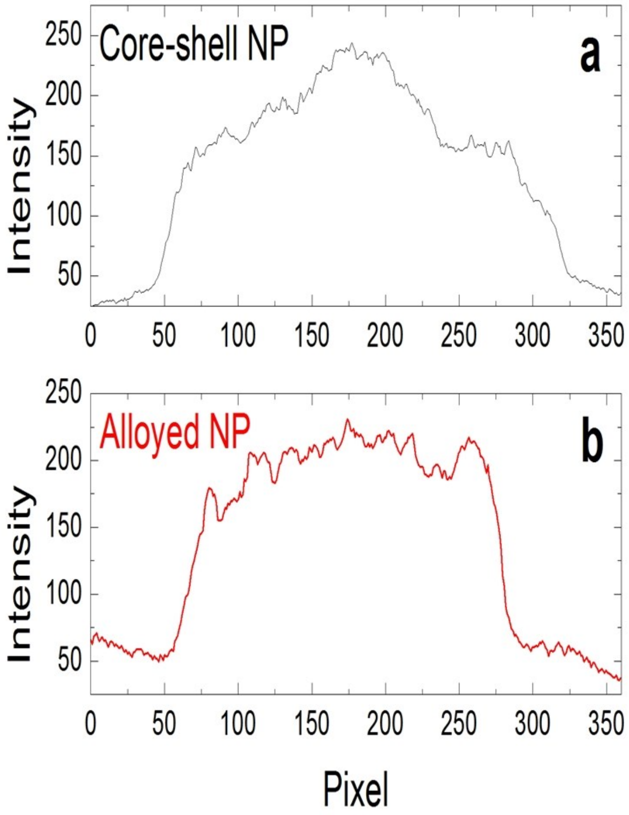

Fig. S4: Profile intensity comparison between core-shell and alloyed particles.

(a) The intensity profile of the core-shell particle shows a non-continuous signal with an increase in the signal at the center. This increase is not due to the thickness but to the presence of more important Z atoms in the center of the particles (here Pt).

(b) The intensity profile of the alloyed particle shows a continuity of the signal due to the thickness of the particle, a homogeneous chemical composition can be deduced within the NP.

3. Alloyed NiPt NPs

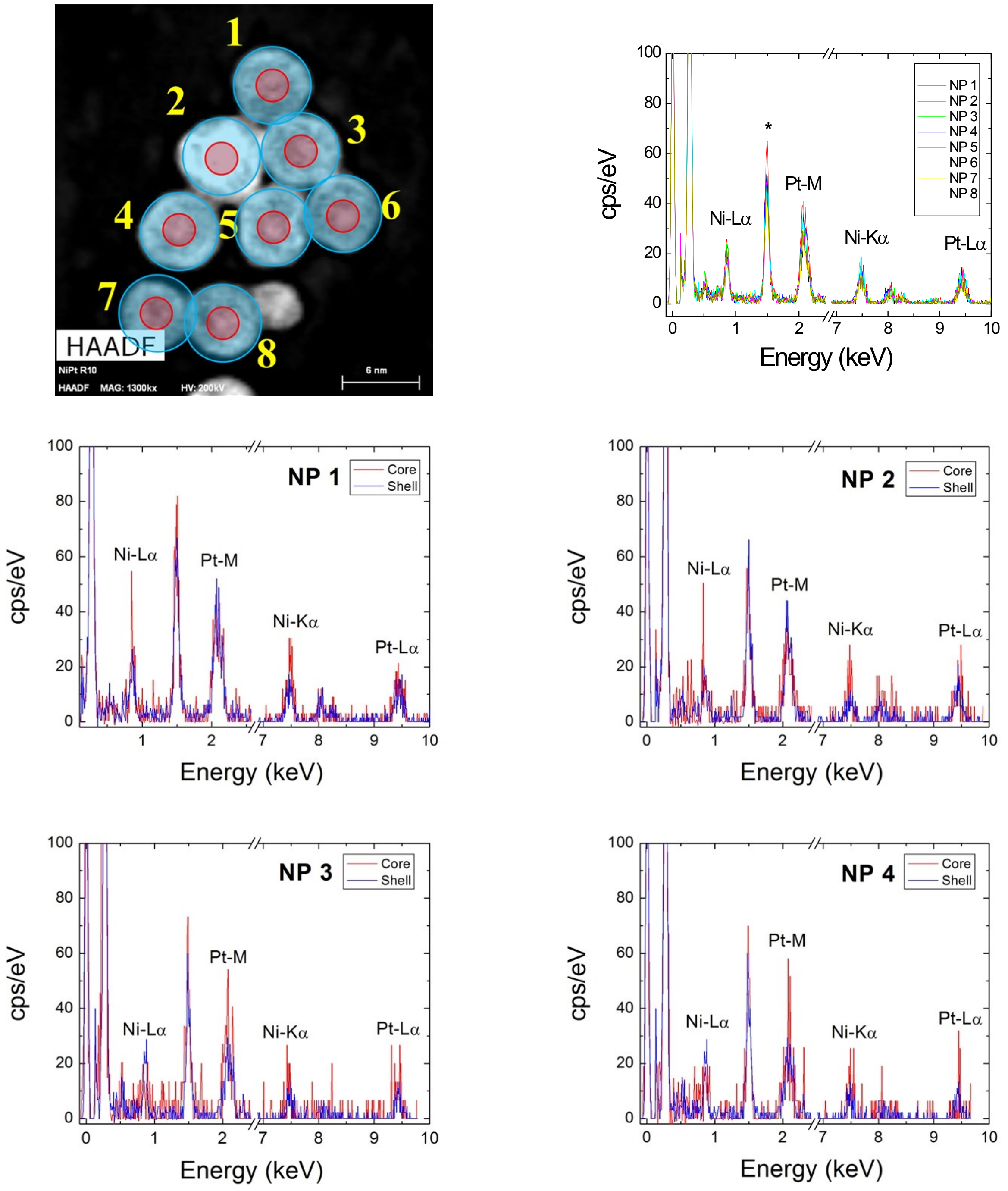

Fig. S5: Analysis of EDX spectra of individual NPs within the NiPt sample

Top HAADF image showing 8 labelled NP of the NiPt sample presented in Fig. 5. Their inner part (core) is highlighted in red and this outer part in blue (shell) and the comparison of the 8 spectra recorded on each NP.

Comparison of the EDX spectra over 8 isolated NPs indicates an identical chemical composition near 56 % in nickel against 44 % in platinum (atomic %).

Bottom comparison for each particle of EDX spectra recorded from the core (red curve) and from their shell (blue curve).
The EDX chemical composition comparison between the inner and outer part of isolated particles attests the composition homogeneity within each NP.
* The aluminum peaks at 1.486 keV is due to the aluminum TEM grid.

4. **Alloyed Ni_xPt_{1-x} NPs**

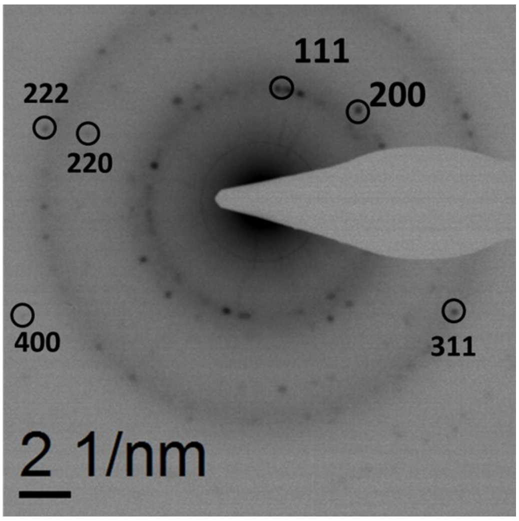

Ni_3Pt sample

In order to have a precise measurement of the lattice parameter, the (hkl) indexing and determination of the FCC lattice parameter was performed on 5 diffractions patterns from 5 different zones on the TEM grid

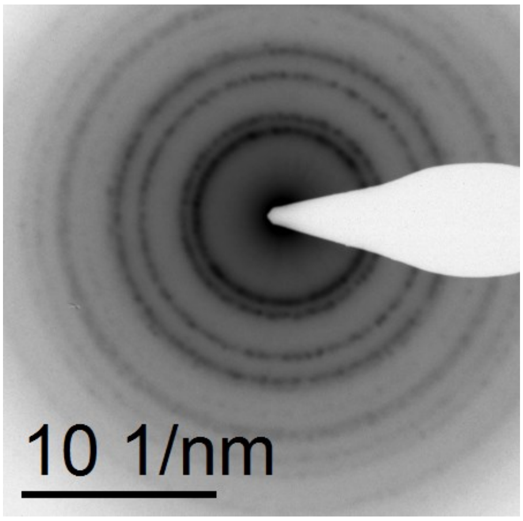

NiPt sample

In this diffraction pattern we note the presence of all (hkl) up to 511, in order :

111, 200, 220, 311, 222, 400, 331, 420, 422, 511

Here again, analysis has been performed on 5 different zones.

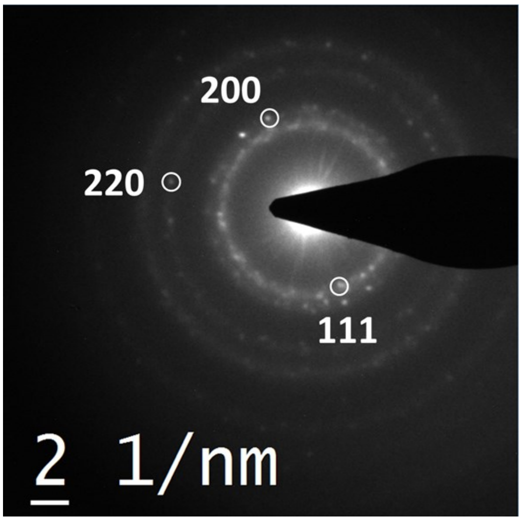

$NiPt_3$ sample

Here to, we note hkl peaks up to 511 but for the sake of understanding the images, not all patterns are indexed and the analysis has been done on 5 different zones.

Fig. S6: Electron diffraction patterns recorded on an assembly of NPs in Ni_3Pt, NiPt and $NiPt_3$ samples (the contrasts are different and have been chosen only for a better visibility).

5. Analyses of PRXD

(hkl)	Ni_3Pt core-shell		NiPt alloy
	NiPt core	Ni_3Pt shell	
**(111)**	0.3706	0.3592	0.3801
**(200)**	0.3749	0.3596	0.3799
**(220)**	_	_	0.3800
**(311)**	_	_	0.3799
**(222)**	_	_	0.3806
**Average**	**0.373 ± 0.003 nm**	**0.359 ± 0.003 nm**	**0.380 ± 0.003 nm**

Table S1: Lattice parameters deduced from peaks indexation of PXRD patterns presented in Fig. 2 (Ni_3Pt core-shell sample) and Fig. 6 (NiPt alloy sample).

$$\tau = \frac{K\lambda}{\beta\cos(\theta)}$$ *Scherrer formula*

With τ corresponding to the mean crystalline domain size, K the shape factor (taken as 0.89), λ the wavelength of the X-ray beam (here λ = 1.541 Å), β the full at half maximum (FWHM) of the diffraction peak and θ the Bragg angle.

6. Heating experiments *in situ*

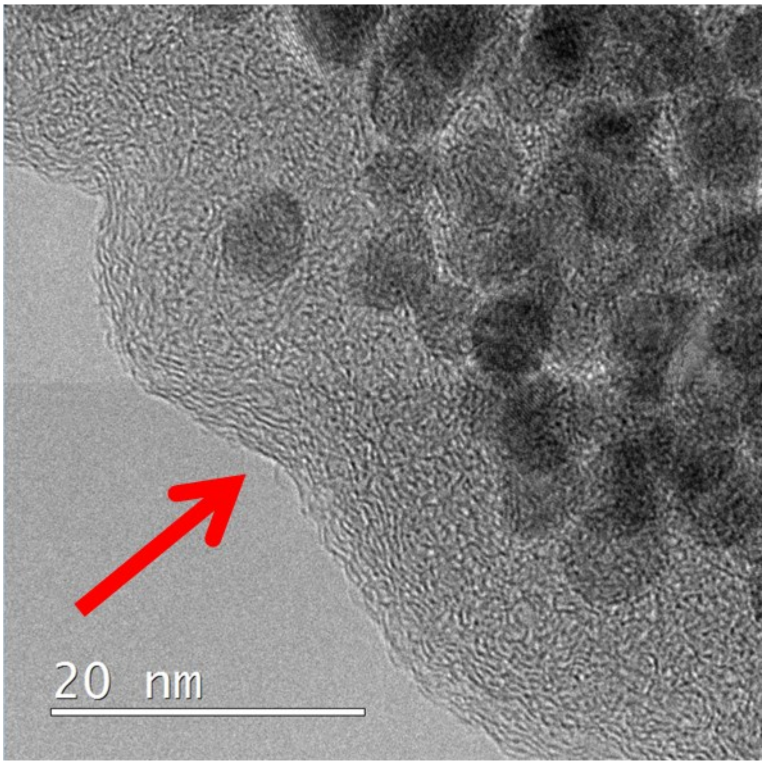

Fig. S7: TEM image of surfactants polymerization at slow heating temperatures.

7. Analyses of electronic diffraction patterns

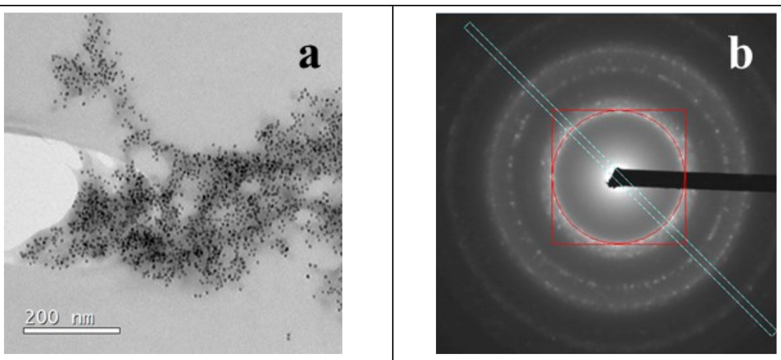

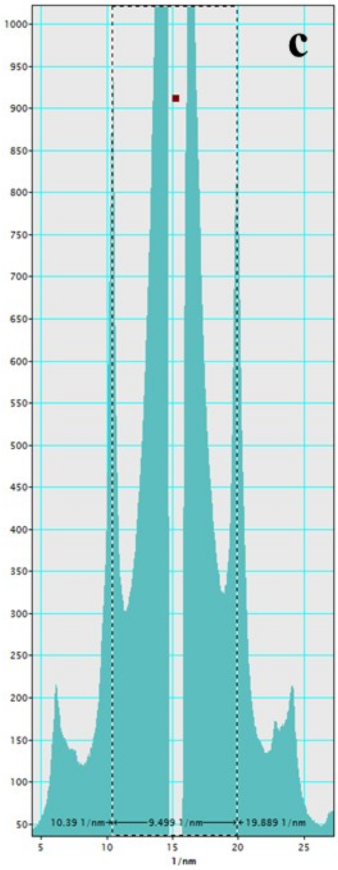

diameter	radius	d*	h	k	l	a* (1/nm)	a (nm)
9.499	4.7495	0.21054848	1	1	1	2.7421251	0.36468066
10.997	5.4985	0.18186778	2	0	0	2.74925	0.36373556
15.441	7.7205	0.12952529	2	2	0	2.72960895	0.36635284
18.16	9.08	0.11013216	3	1	1	2.73772301	0.36526705
18.617	9.3085	0.10742869	2	2	2	2.68713249	0.37214391

For each fringe, we take the maximum of the intensity thanks to the intensity profile obtained on the blue line on electron diffraction pattern. Then, we calculate the lattice parameter for each observable atomic plane. Finally, we average the different "**a**" obtained and we calculate the standard deviation which is

our error bar. Here, a = (0.366 ± 0.003) nm.	

Table S2: Methodology to determine the lattice parameters and its error bar with (**a**) TEM image of the diffracted zone, (**b**) diffraction pattern with blue line corresponding to the intensity profile through the center of the diffraction pattern (the red circle and the red square are traced to find the center), (**c**) the intensity profile and Excel table to calculate the lattice parameter deduced by the circle diffraction diameters.

**Microscope**	**Micrographies**	**Electron diffraction patterns**	**Lattice parameters (nm)**
**ZEISS-LIBRA 200 MC**	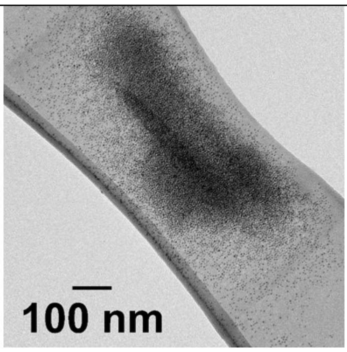	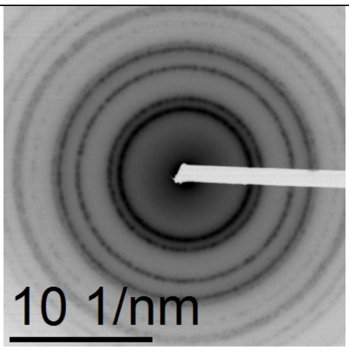	0.377 ± 0.003
**JEOL 1400**	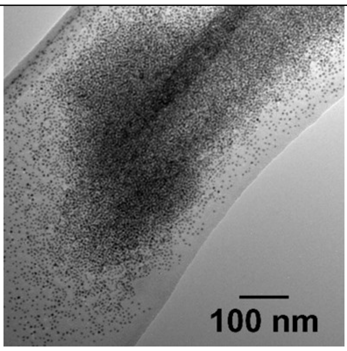	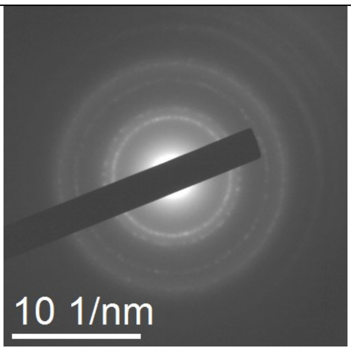	0.380 ± 0.003

**FEI-CM 20**	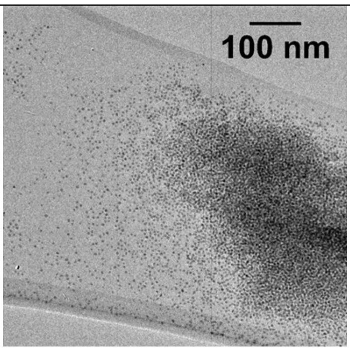	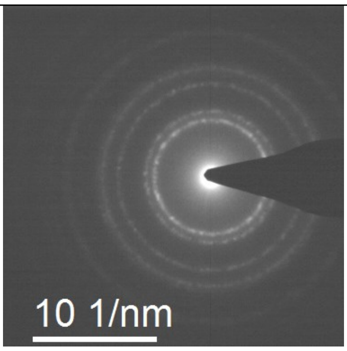	0.382 ± 0.001

Table S3: Lattice parameter comparison on the same TEM grid zone of NiPt (56:43) sample with three different microscopes; Zeiss-Libra 200 MC, Jeol 1400 and FEI-CM 20.

**Microscope**	**Micrographies**	**Electron diffraction patterns**	**Lattice parameters (nm)**
**ZEISS-LIBRA 200 MC**	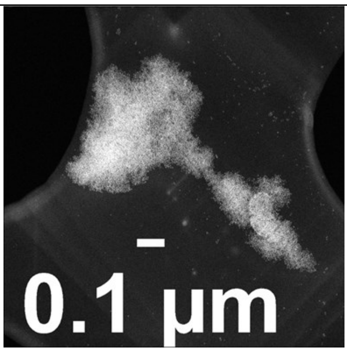	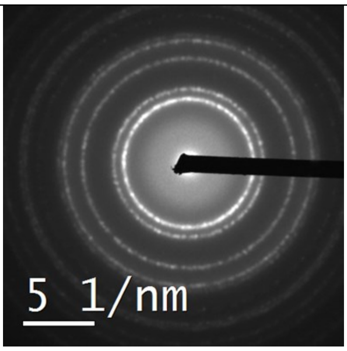	0.384 ± 0.007
**JEOL 1400**	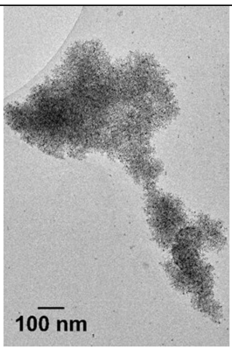	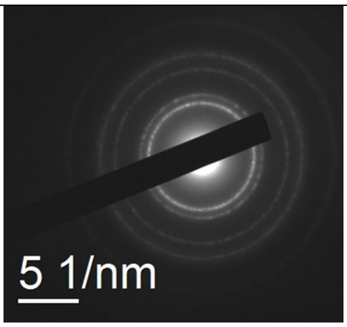	0.383 ± 0.002

**FEI-CM 20**	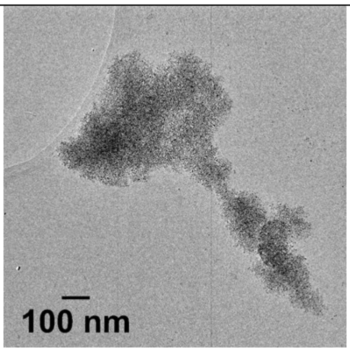	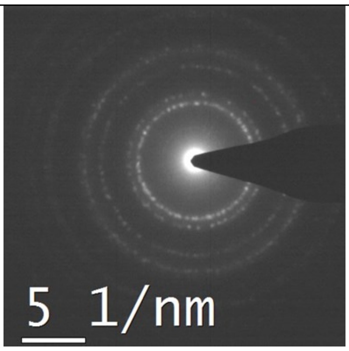	0.382 ± 0.002

Table S4: Lattice parameter comparison on the same TEM grid zone of $NiPt_3$ (30:70) sample with three different microscopes; Zeiss-Libra 200 MC, Jeol 1400 and FEI-CM 20.
